# Everyday discrimination and cognitive function among older Latino adults

**DOI:** 10.1093/geroni/igaf122.3758

**Published:** 2025-12-31

**Authors:** Mayra Estrella, Maude Wagner, Lisa Barnes, Brittney Lange-Maia, David Marquez, Melissa Lamar

**Affiliations:** Rush University Medical Center, Chicago, Illinois, United States; Rush University Medical Center, Chicago, Illinois, United States; Rush University Medical Center, Chicago, Illinois, United States; Rush University Medical Center, Chicago, Illinois, United States; University of Illinois Chicago, Chicago, Illinois, United States; Rush University Medical Center, Chicago, Illinois, United States

## Abstract

Greater exposure to everyday discrimination is associated with poorer cognitive health among older Black adults; however, its role in cognitive function among older Latino adults remains largely unexplored. This study examined associations of self-reported experiences of everyday discrimination with cognitive level and change among older Latino adults. We included 351 Latino participants (age=71±7yrs; 79% female) from Rush Alzheimer’s Disease Center cohort studies without clinical dementia at baseline and who completed ≥2 cognitive evaluations over time (mean follow-up=7±4yrs). Everyday discrimination was assessed at baseline using the Detroit Area Study Everyday Discrimination Scale (range=0-9). Separate linear mixed-effects models tested associations of everyday discrimination with initial level and annual rates of change in global cognition and five cognitive domains (episodic, working, and semantic memory; perceptual speed and visuospatial ability), adjusting for age at baseline, sex, education, and preferred language (English/Spanish) for testing. Higher levels of everyday discrimination were associated with worse initial global cognition (β=-0.048, Standard Error [SE]=0.012, p = 0.0001), and separate domains including episodic memory (β=-0.042, SE = 0.015), semantic memory (β=-0.047, SE = 0.017), working memory (β=-0.078, SE = 0.017), and perceptual speed (β=-0.066, SE = 0.018; all P < 0.01). There were no associations with cognitive decline. Findings extend previous evidence from cohorts of older Black adults to older Latino adults showing that greater everyday discrimination is associated with worse initial global cognition and most cognitive domains, but not with rates of cognitive decline. As baseline cognitive level may shape dementia risk, this study underscores the importance of addressing social exposures among Latinos to support healthy cognitive aging.

